# Prevalence of Positive COVID-19 Test Results Collected by Digital Self-report in the US and Germany

**DOI:** 10.1001/jamanetworkopen.2022.53800

**Published:** 2023-01-31

**Authors:** Jakob J. Kolb, Jennifer M. Radin, Giorgio Quer, Annika H. Rose, Jay A. Pandit, Marc Wiedermann

**Affiliations:** 1 Robert Koch Institute, Berlin, Germany; 2 Scripps Research Translational Institute, La Jolla, California

## Abstract

This cohort study examines traditional surveillance and self-reported COVID-19 test result data collected from independent smartphone app–based studies in the US and Germany.

## Introduction

Since the beginning of the COVID-19 pandemic, official case numbers, typically collected in health care settings, have been a guideline for public health policies. As rapid home testing has become increasingly available, more people have been able to self-diagnose SARS-CoV-2 infections at home, which is not typically counted in official statistics. Here, we aimed to evaluate the agreement and discuss potential sources of deviation between traditional surveillance and self-reported COVID-19 test result data collected from independent smartphone app–based studies in the US and Germany.

## Methods

The US-based Digital Engagement and Tracking for Early Control and Treatment (DETECT) study and the Germany-based Corona-Datenspende (CDA) allow participants to complete an eConsent, answer surveys about COVID-19 symptoms, testing, and vaccination, and to donate sensor data through a smartphone app (see eMethods in Supplement 1). We compared the 7-day rolling mean values of self-reported COVID-19 positive swab test results in both data sets during our observation period (see Table) together with respective 7-day rolling averages of case counts (US Centers for Disease Control^1^) and incidences (Robert Koch Institute, Germany^2^). Statistical analysis was performed from March 2020 to July 2022 for DETECT and from January 2022 to July 2022 for CDA using Python 3.10 with pandas 1.4.3 (both community-maintained open source). This cohort study followed the STROBE reporting guideline.

**Table.** Summary of DETECT and CDA

Variable	Participants, No. (%)	
	DETECT	CDA
Date study started	March 2020	April 2020/October 2021^a^
Observation period	March 2020 to July 2022	January 2022 to July 2022
No. of participants in observation period	40 646	24 017
Participant characteristics		
Age, mean (SD), y	49.4 (15.1)	48.1 (14.2)
Gender		
Female	24 834 (61.1)	8633 (35.9)
Male	15 585 (38.3)	13030 (54.2)
Other	159 (0.4)	46 (0.0)
NA^b^	68 (0.2)	2308 (9.6)
COVID-19 testing^c^		
Positive	3830 (10.9)	12 790 (6.9)
Negative	31247 (89.1)	171 430 (93.1)

Abbreviations: CDA, Corona-Datenspende; DETECT, Digital Engagement and Tracking for Early Control and Treatment.

^a^ Parent study launched in April 2020, surveys were available from October 2021.

^b^ In CDA, users were not required to specify their gender and for those who did not, gender is NA (not available).

^c^ Swab test results include polymerase chain reaction and antigen tests.

## Results

During the observation period for DETECT (March 2020 to July 2022), 40 646 participants were enrolled who self-reported 35 077 COVID-19 test results; and during the observation period for CDA (January 2022 to July 2022), 24 017 participants were enrolled who self-reported 184 220 COVID-19 test results. Both studies had a similar mean (SD) participant age (49.4 [15.1] years in DETECT and 48.1 [14.2] years in CDA), but DETECT had a higher proportion of female participants (61.1% [n = 24 834]) compared with CDA (35.9%% [n = 8633]).

In both studies, self-reported cases correlated well with official numbers (Pearson correlation coefficient of 0.70 for CDA and 0.75 for DETECT) and onsets and turning points of pandemic waves are well aligned (Figure). However, we noted a stagnation of self-reported cases in the US in October 2021 and in Germany in February and March 2022 when case numbers were still on the rise. Likewise, self-reported positive tests pick up at larger rates in April 2022 (US) and June 2022 (Germany) compared with the official case numbers, even though the respective peaks of the waves are still estimated around the same time points.

**Figure.**
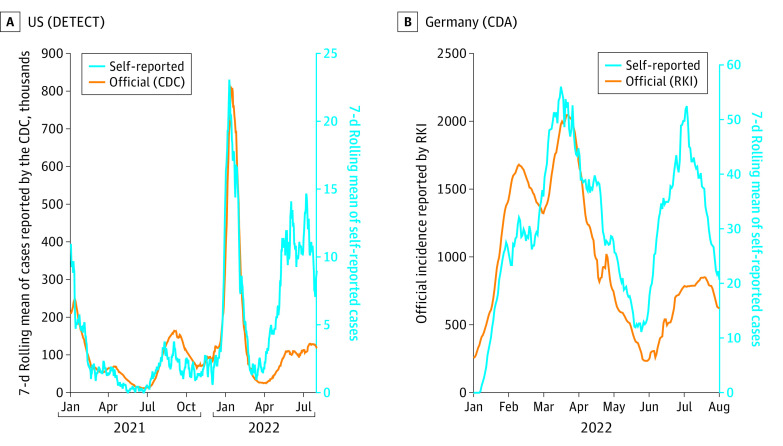
Official 7-day Moving Mean of COVID-19 Cases Compared With the 7-day Rolling Mean of Self-reported Infections in the US and Germany Orange lines are data reported through official channels, blue lines are self-reported data. DETECT: March 2021 to July 2022; CDA: January 2022 to July 2022. CDC indicates US Centers for Disease Control and Prevention; RKI, Robert Koch Institute.

## Discussion

Diagnostic testing for COVID-19 infections has seen a shift from polymerase chain reaction tests in health care settings toward rapid antigen testing done at home. If people do not follow up on a positive home test with one taken in a health care setting, they might not be captured by established surveillance systems, which could partially explain some of the observed deviations between self-reports and official statistics. Hence, digital tools such as DETECT and CDA have the potential to provide real-time complementary data to further inform policy.

However, the crowdsourced data used in this study comes with a set of limitations. Both studies exclude children and adolescents and show a strong underrepresentation of older people. Furthermore, participants might be particularly health aware and more risk averse than the general population. This might partially explain the large increase in self-reported cases in later phases of the pandemic when infections became increasingly difficult to avoid. Furthermore, both studies saw declining participation over time, which might lead to more erroneous estimations of COVID-19 prevalence from self-reported data for more recent waves. Individuals possibly also tested multiple times during their infection and therefore the absolute numbers of cases were overcounted. Ultimately, antigen tests are less sensitive compared with polymerase chain reaction tests in early and late stages of infection, which should also be considered before operationalizing such crowdsourced systems in the future.

Prior digital crowdsourcing platforms such as Outbreaks Near Me (in the US) and GrippeWeb (in Germany) have shown their ability to track flu-like illnesses^3^ in real-time. As with DETECT^4^ and CDA,^5^ they have some unique advantages, including scalability, location independence, minimal time and effort from participants, adaptability, and low cost. Similar to wastewater monitoring,^6^ they can complement clinical (in-person) surveillance by tracking the magnitude of COVID-19 activity when cases might become increasingly diagnosed at home and thus may not all be captured by public health counts.

## Notes

Supplement 1. **eMethods.** Supplemental Methods

Supplement 2.
Data Sharing Statement
