# Supplementary material for: Prevalence of Positive COVID-19 Test Results Collected by Digital Self-report in the US and Germany
Source: JAMA Netw Open. 2023 Jan 31;6(1):e2253800. doi: 10.1001/jamanetworkopen.2022.53800 (PMC9890282; doi:10.1001/jamanetworkopen.2022.53800)
Supplement: Supplement 1. — eMethods. Supplemental Methods [file jamanetwopen-e2253800-s001.pdf]

Supplemental Online Content

Kolb JJ, Radin JM, Quer G, Rose AH, Pandit JA, Wiedermann M. Prevalence of positive COVID-19 test results collected by digital self-report in the US and Germany. *JAMA Netw Open.* 2023;6(1):e2253800. doi:10.1001/jamanetworkopen.2022.53800

eMethods. Supplemental Methods

This supplemental material has been provided by the authors to give readers additional information about their work.

© 2023 Kolb JJ et al. *JAMA Network Open.*

eMethods. Supplemental Methods

Prevalence of Positive COVID-19 Test Results Collected by Digital Self Report in the U.S. and Germany

Participant recruitment and eligibility

DETECT: Participation was voluntary. Participants were eligible if they were 18 years or older, lived in the US and downloaded the MyDataHelps app.

CDA: Participation was voluntary, self-recruited and is only possible for German residents age 16 and older.

Apps for both studies are available for Android and iOS.

Access to Home Testing

In our study, we saw drastically higher testing frequency in Germany compared to the United States. In the US, rapid home tests can be purchased at retail pharmacies for ~$20 and limited numbers have been provided by the U.S. Government and insurance companies. In Germany, tests have, among others, been required for entry to schools or public events and only cost $1-2.

Data Collection

DETECT: Participants are given weekly push notifications through the app to remind them to fill out surveys if they had any new symptoms, test results, test type (swab test, saliva, or blood), or vaccinations. The option to fill out these surveys was voluntary and likely captured mostly new COVID-19 cases as they occurred, making the numbers closer to incidence rather than prevalence.

CDA: Every seven days, enrolled participants received a push notification asking them to open the app and provide information whether a COVID-19 test was taken any time during the past seven days. If so, participants are then asked to provide test type (PCR, antigen or antibody) and result (positive or negative). Participation was voluntary, so participants could also choose to not enter any information. In that case, a new push notification was sent 7 days later. In Germany, during the observation period, it was required to provide a negative test to end mandatory quarantine. It is therefore likely that participants reported positive test results for each consecutive week of their infection and thus the 7 day rolling average of positive reported tests reported in the CDA is a proxy for the prevalence of infections in the cohort of participants.

Ethical Approval

The DETECT Study was reviewed and approved by the Scripps Office for the Protection of Research Subjects (Institutional Review Board 20-7531). The study protocol can be found here:

© 2023 Kolb JJ et al. *JAMA Network Open.*

The DETECT(Digital Engagement & Tracking for Early Control, & Treatment) Study - Full Text View - ClinicalTrials.gov

The Corona Data donation project was approved by the ethics board at the University of Erfurt (approval number 20220414).

© 2023 Kolb JJ et al. *JAMA Network Open.*
