# Supplementary material for: Prevalence of Positive COVID-19 Test Results Collected by Digital Self-report in the US and Germany
Source: JAMA Netw Open. 2023 Jan 31;6(1):e2253800. doi: 10.1001/jamanetworkopen.2022.53800 (PMC9890282; doi:10.1001/jamanetworkopen.2022.53800)
Supplement: Supplement 2. — Data Sharing Statement [file jamanetwopen-e2253800-s002.pdf]

Data Sharing Statement

Kolb. Prevalence of Positive COVID-19 Test Results Collected by Digital Self-Report in the US and Germany. *JAMA Netw Open*. Published January 31, 2023.
doi:10.1001/jamanetworkopen.2022.53800

Data
Data available: No

Additional Information
Explanation for why data not available: We will make the original data available eventually, but are currently in the process of defining a GDPR compliant way to do so together with the respective data privacy officers.
